# Role of *Spirulina platensis* and Humic Acid in Mitigating Acute Cyclic Heat Stress: Effects on the Growth Performance, Meat Quality, Immunological Responses, and Tissue Histomorphology in Broiler Chickens

**DOI:** 10.3390/vetsci12121187

**Published:** 2025-12-12

**Authors:** Shimaa A. Amer, Ahmed Gouda, Rehab I. Hamed, Abdel-Wahab A. Abdel-Warith, Elsayed M. Younis, Arwa H. Nassar, Hanaa S. Ali, Rania M. Ibrahim, Mona S. Ibrahim, Shereen Badr, Simon J. Davies, Gehan K. Saleh

**Affiliations:** 1 Department of Nutrition & Clinical Nutrition, Faculty of Veterinary Medicine, Zagazig University, Zagazig 44511, Egypt; 2 Animal Production Department, Agricultural & Biological Research Division, National Research Center, Dokki, Cairo 11865, Egypt; 3 Department of Poultry Diseases, Reference Laboratory for Quality Control on Poultry Production (RLQP), Animal Health Research Institute (AHRI) (Zagazig Branch), Agriculture Research Center (ARC), Zagazig 44516, Egypt; 4 Department of Zoology, College of Science, King Saud University, P.O. Box 2455, Riyadh 11451, Saudi Arabia; 5 Food Hygiene Department, Animal Health Research Institute (AHRI) (Mansoura Branch), Agriculture Research Center (ARC), P.O. Box 246, Dokki, Giza 12618, Egypt; 6 Department of Pathology, Animal Health Research Institute (AHRI) (Mansoura Branch), Agriculture Research Center (ARC), P.O. Box 246, Dokki, Giza 12618, Egypt; 7 Department of Poultry Diseases, Animal Health Research Institute (AHRI) (Mansoura Branch), Agriculture Research Center (ARC), P.O. Box 246, Dokki, Giza 12618, Egypt; 8 Department of Clinical Pathology, Animal Health Research Institute (AHRI) (Mansoura Branch), Agriculture Research Center (ARC), P.O. Box 246, Dokki, Giza 12618, Egypt; 9 Aquaculture Nutrition Research Unit (ANRU), Carna Research Station, Ryan Institute, College of Science and Engineering, University of Galway, H91V8Y1 Galway, Ireland; 10 Biochemistry Department, Animal Health Research Institute (AHRI) (Mansoura Branch), Agriculture Research Center (ARC), P.O. Box 246, Dokki, Giza 12618, Egypt

**Keywords:** poultry, dietary addition, stress, growth, immunity, meat quality

## Abstract

Elevated ambient temperature is the primary abiotic element that possibly diminishes production and economic profitability in the chicken industry. The current study evaluated the role of dietary addition of *Spirulina platensis* (SP) and humic acid (HA) in mitigating the effects of acute cyclic heat stress on growth performance, meat quality, immune status, and intestinal morphology in broiler chickens. Five hundred three-day-old male Ross 308 broiler chicks (average body weight 101.42 ± 3.22 g) were assigned to five experimental groups, each with 10 replicates (10 birds per replicate). The chicks in the first group received a basal diet and were maintained in thermoneutral conditions (NEG CON). The remaining four groups received either a basal diet only (POS CON) or a basal diet added with SP (2 g/kg of feed; SP group), HA (5 g/kg of feed; HA group), or a mix of SP and HA by the same doses (SP+HA group). The four groups were exposed to acute cyclic heat stress (36 °C ± 2 °C) from the 22nd to the 25th day of age for 6 h/day. The HA group showed increased body weight and body weight gain, and improved feed conversion ratio compared with other groups (*p* < 0.001). The addition of SP and HA improved sensory characteristics and reduced the dripping losses of the breast muscles. The phagocytic % and phagocytic index were higher in the SP group compared with the NEG and POS CON. The serum levels of triiodothyronine and thyroxine were higher in the HA and SP+HA groups compared with the control groups. The serum concentrations of interleukin-10, complement 3, and lysozymes, as well as the liver concentrations of HSP90A and HSP90B, were higher in the SP, HA, and SP+HA groups compared with the NEG and POS CON. The duodenal villous height and width were significantly greater in the HA group compared to the other groups. Spleen histomorphology in the SP and HA groups was better than that of the POS CON. The HA group showed up-regulation in the immune expression of clusters of differentiation 3 (CD3) and 20 (CD20) proteins in the spleen tissues. In conclusion, both HA and SP, individually but not in combination, mitigated the detrimental effects of acute cyclic heat stress on growth and immunity. Humic acid addition provided the most pronounced improvements in performance and intestinal morphology. Further studies are warranted to clarify the biochemical interactions between SP and HA under different stress intensities.

## Simple Summary

Heat stress is detrimental to poultry production, welfare, and health. Several strategies have been employed to mitigate these consequences. The current study assessed the role of dietary addition of *Spirulina platensis* (SP) and humic acid (HA) in alleviating the effects of acute cyclic heat stress. The study’s outcomes showed that the single addition of either HA or SP effectively attenuated the detrimental effects of acute cyclic heat stress on growth, meat quality, metabolism, and immunity. The single additions of SP or HA were more effective than their combination, with HA producing the greatest improvement in growth performance and intestinal morphology.

## 1. Introduction

Elevated ambient temperature is the primary abiotic element that possibly diminishes production and economic profitability in the chicken industry. Seasonal fluctuations in temperature and relative humidity (RH) can lead to extreme conditions. Variations in environmental temperature, both below and above the thermal comfort zone, adversely affect broiler performance, resulting in diminished growth rates, immunological suppression, vaccine failures, and irreparable organ damage [1,2,3]. In addition to influencing protein synthesis and absorption [4], exposure to heat stress (HS) enhances water uptake while reducing feed intake in broilers [5,6]. Heat stress impairs gastrointestinal integrity, compromising nutrient digestion and absorption. It also adversely affects immunology, microbiology, and physiology, leading to compromised and aberrant gastrointestinal function in birds [7].

Acute heat stress (HS) occurs when birds are exposed to high temperatures that exceed the thermal requirements of chickens for a brief period (a few hours to a few days) [8]. In contrast, chronic HS occurs when temperatures are continuously high for an extended period (days to months) [9]. Despite having a relatively low severity, chronic HS is more common and persists for extended periods, often recurring more frequently in tropical regions [10]. Climate-resilient poultry farming, which primarily relies on effective management techniques, is crucial for achieving maximum productivity in tropical nations. The optimal temperatures at which the bird can function most effectively are determined by its age, body weight, housing system, feeding level, relative humidity, air velocity, and overall health [11].

Evaporative heat dissipation is a crucial mechanism significantly affected by the surrounding environment’s relative humidity. Increased humidity diminishes evaporative heat loss, which escalates with temperature. The impact of humidity on the thermal regulation response of broiler chickens is influenced by age and air temperature [12]. The effect of humidity on the performance of broiler chicks and turkeys exposed to temperatures as high as 28 °C and 30 °C, respectively, is substantial [13]. Humidity affected the thermoregulation of 1-week-old broiler hens by redistributing heat throughout the body at high temperatures, causing the peripheral temperature to rise and the rectal temperature to fall [12].

Finding internal solutions to lessen the negative impacts of HS would be beneficial. Various dietary techniques were employed to enhance the health of heat-stressed broilers, including the use of phytogenic feed additives, polyphenols, essential oils, minerals, vitamins, and beneficial bacteria [14]. Natural feed additives, like algal biomass or extracts that are high in antioxidants and other bioactive components, may provide two benefits in such circumstances, like heat stress: first, they may help birds withstand unsuitable or stressful environmental conditions; second, they may produce broilers with lower levels of cholesterol or total lipids and likely better antioxidant status [15]. Microalgae are a cost-effective and sustainable source of biofuels, bioactive pharmaceuticals, and feed ingredients [16]. Chlorella species and Spirulina (*Arthrospira platensis*) are among the most robustly utilized microalgae for feed and nutrition. The blue-green algae, known as spirulina (SP), is one of nature’s most adaptable products, offering numerous benefits for human and animal health. Spirulina is enhanced with a high protein content, vitamins, xanthophyll colors, and many vital amino acids. It is also enriched with high quantities of β-carotene, vitamin B12, iron, and trace minerals, as well as γ-linolenic acid [17]. Numerous cyanobacteria strains are known to generate extracellular and intracellular compounds with antibacterial [18] and immune-stimulating [19] properties. Furthermore, there were reports of antiplatelet [20], anticardiotoxic [21], antinephrotoxic [22], antihepatotoxic [23], and hypocholesterolemic [24] effects. The in-feed benefits of SP in boosting immunity and reducing oxidative stress in broilers exposed to HS at a 2% level were previously studied [15,25]. It has also been shown that in-feed SP can improve broiler meat quality [26] and serve as a substitute for soybean meal [27].

Humic acid, fulvic acid, and trace minerals make up humic substances, also known as humates, which include reed sedge peat compounds. The improvement in weight gain and enhanced feed conversion may be attributed to beneficial effects on metabolic processes of digestion and nutrient utilization; however, humate addition does not enhance growth by altering feed intake [28]. In other animal species, humates have also been linked to improved health through physiological alterations and enhanced immune development [29]. Humic compounds are utilized as natural growth boosters due to their antibacterial, detoxifying, antifungal, and antioxidant properties [30]. It could lower several types of stress and enhance the immune system of birds [31]. In broilers, Gomez-Rosales and de L Angeles [32] discovered that HA enhanced nutrient retention and ileal digestibility of energy. Due to the benefits mentioned above, SP and HA were evaluated for their potential to mitigate the effects of acute cyclic heat stress on broiler chicks. Many previous studies have demonstrated the beneficial effects of individual addition of SP and HA on growth, immunity, and oxidative stress. To our knowledge, no studies have assessed the impact of combining SP and HA on alleviating acute cyclic heat stress in broiler chickens. We hypothesize that there would be a synergism between SP and HA that can beneficially mitigate the effects of acute heat stress.

## 2. Materials and Methods

### 2.1. Feed Additives

*Arthrospira platensis* (SP) was obtained from the Algal Biotechnology unit, National Research Centre, Egypt. According to El-shennawy, et al. [33], total flavonoid content was 84.5 mg/100 g, the total phenolic content was 750.8 mg/100 g, total carotenoids were 1320 mg/100 g, and phycocyanin was 1780 mg/100 g [33]. Humic acid powder (HA) was obtained from Humin Tech, Grevenbroich, Germany. The product contains 496 mmol (eq)/100 functional groups, including 260 carboxyl and 236 phenolic groups with pKa 4.5, 6.5, and 9.5.

### 2.2. Birds, Experimental Planning, and Diets

The experiment was conducted at the Avian Experimental Centre of Zagazig University’s Faculty of Veterinary Medicine in Egypt. All experiment procedures were approved by the ARC-IACUC committee (Approval No. ARC-IACUC/AHRI/152/24).

A local hatchery provided 500 one-day-old male Ross 308 broiler chicks. They were reared for a 35-day feeding trial. On the first 3 days, they were exposed to a pre-experiment adaptation period till they reached an average starting weight of 101.42 ± 3.22 g. During the adaptation period, the chicks received a broad-spectrum antibiotic (oxytetracycline, 20% (1 g/L) in drinking water) and were fed the control starter diet. Then, they were randomly assigned to five experimental groups, each with 10 replicates (10 birds per replicate). In the first group, the chicks received a basal diet and were maintained in thermoneutral conditions (NEG CON). The remaining four groups received either a basal diet only (POS CON) or a basal diet added with SP (2 g/kg of feed; SP group) [34], HA (5 g/kg of feed; HA group) [35], or a mix of SP and HA by the same doses (SP+HA group). The four groups were exposed to acute cyclic heat stress. As advised for ROSS broiler chicks, the standard brooding temperature was initially raised to 34 °C and then gradually lowered to 23 °C by the end of the rearing period [36]. The average relative humidity ranged from 50% to 55% in the CON NEG group. From the 22nd to the 25th day of the feeding period, the groups that experienced acute cyclic heat stress were maintained at an ambient temperature of 36 ± 2 °C for six hours per day (11:00 a.m.–5:00 p.m.). The relative humidity ranged from 68% to 70%. A temperature-humidity index (THI) ranged from 38 to 41% (Table 1); subsequently, the temperature decreased to the level of the CON NEG group for the remainder of the experiment. A heater-air conditioning system was used to keep the temperature within this range. The THI was consistently determined using the formula from the World Meteorological Organization [37]: THI = Td − [0.55 − (0.55 × RH/100)] × (Td − 58). Where Td is the dry bulb temperature in degrees Celsius. For the first three days of the experiment, the lighting system in each experimental pen was maintained at 23:1 h light: dark, and thereafter, it was maintained at 20:4 h light: dark until the end of the experiment. During the study, birds had unrestricted access to feed and water, and diets were provided as mash. The chicks were raised in an outside, well-ventilated housing with suitable litter. The chicks were fed on two experimental diets. Diet A for the NEG CON, POS CON, and HA-added groups. Diet B for the SP-added group and HA+SP-added group (Table 2). According to AVIAGEN’s advice, immunization schedules, illumination, and dietary formulations were implemented [36]. No mortalities were recorded during the study.

### 2.3. Growth Indices

The feeding period was divided into three periods: starter (4th–10th day), grower (11th–23rd day), and finisher (24th–35th day). On the fourth day of their lives, the chicks were weighed. The average weight at various feeding stages was then estimated by recording body weights at 10, 23, and 35 days.

Body weight gain (BWG) = W2 − W1. W1 is the weight at the beginning of the scheduled time, and W2 is the weight at the end of that period.

The weight of the feed supplied was deducted from the leftovers for each replicate, and the result was divided by the number of birds in that replicate to determine the average feed intake (FI) per bird.

Feed conversion ratio (FCR) was calculated as FI (g) divided by BWG (g).

### 2.4. Sampling

Ten birds were randomly chosen for tissue and blood collection from each group. On day 35 of the trial, they were euthanized by cervical dislocation following an 8 h fast [38]. Two portions of blood samples were collected; the first was collected into heparinized tubes to determine the hematological parameters and phagocytic activity. The latter was collected into 3 mL vacutainer tubes, centrifuged for 10 min at 1006× *g*, and the resulting serum was stored at −20 °C until used in biochemical testing. Breast muscle samples (*n* = 10) were removed and chilled for 5–6 h at 4 °C to evaluate the meat quality measures. Another set of meat samples (*n* = 10) was vacuum-packed and stored at −20 °C to determine the proximate chemical and fatty acid composition. After that, samples (*n* = 10) were taken from the small intestine (duodenum, jejunum, and ileum) and spleen for histological examination. Additional splenic samples (*n* = 10) were extracted for immunohistochemical examination. Liver samples (*n*= 10) were taken to analyze heat shock protein 90 (HSP90A and HSP90B).

### 2.5. Meat Quality, Fatty Acid, and Chemical Composition of the Breast Muscle

The sensory characteristics (color, consistency, and odor) of the muscles under examination were assessed by a trained ten-person descriptor panel, which then assigned a score on a scale of 1 to 5, where 5 denotes normal, 4 denotes slight deviation, 3 denotes moderate deviation, 2 denotes major deviation, and 1 denotes severe deviation. The breast muscles were sampled from the carcasses to assess pH, thawing losses, and cooking (samples were taken for 10 min to attain 75 °C in a preheated water bath) according to Petracci and Baéza [39]. Water-holding capacity (WHC) was measured by centrifuging 4× *g* of breast muscle for 4 min at 1500× *g* in a filter paper (Hettich Laborgeräte GmbH & Co. KG, EBA 200, Tuttlingen, Germany). After that, the samples were dried for 24 h at 70 °C in an oven. The following formula was used to determine WHC [40]:

WHC = [Weight after centrifugation (g) − weight after drying (g)]/Initial weight (g) × 100

At the trial’s conclusion, ten breast muscle samples were taken from each group. The oils from the breast muscle were extracted using a solvent of chloroform/methanol (2:1, *v*/*v*) [41]. The chemical analysis of the breast muscle (dry matter, fat, crude protein, and ash content percent) and the fatty acids in the extracted oil were determined [42].

### 2.6. Hematological Parameters

Total erythrocytes (RBCs), white blood cells (WBCs), and differential leucocytic counts were measured using a Hemascreen 18 Automatic Cell Counter (Hospitex Diagnostics, Sesto Fiorentino, Italy) in accordance with the manufacturer’s instructions [43]. A colorimetric kit (BioDiagnostic, Giza, Egypt) was used to measure the hemoglobin (Hb) concentration using the cyanmethemoglobin approach, as previously reported [44]. Following centrifugation of blood samples in Wintrobe hematocrit tubes (2000× *g*, 20 min, and 4 °C), hematocrit (HT) ratios were calculated by measuring the packed cell volume on a graduated scale. The following formulas were then used to estimate the mean corpuscular volume (MCV), mean corpuscular hemoglobin (MCH), and MCH concentration (MCHC):

MCV (fL) = HT (%)/RBC (10^6^/µL) × 10

MCH (pg) = HB (g/dL)/RBC (10^6^/µL) × 10

MCHC (%) = HB (g/dL)/HT (%) × 100

### 2.7. Phagocytic Activity

White blood cells (WBCs) were isolated from peripheral blood using Ficoll-Histopaque density gradient centrifugation to determine phagocytic activity [45]. The phagocytic index was determined as described by Omar, et al. [46].

### 2.8. Thyroid Hormones, Immune Status Indices, and Heat Shock Proteins

Thyroxine (T4) and triiodothyronine (T3) were measured using chicken ELISA kits from My Biosource Co., San Diego, CA, USA, with CAT. NO., MBS265796 and MBS269454, respectively, per the instructions in the pamphlets.

The chicken-specific ELISA kits (My BioSource Co., CAT.NO. MBS701683 and Life Span Biosciences, Inc., Seattle, WA, USA, CAT. NO. LS-F9287) were used to evaluate the serum levels of interleukin-10 (IL-10) and complement-3 (C3). The method of Lie, et al. [47] was used to measure the serum’s lysozyme activity.

To measure heat shock protein 90 (HSP90A and HSP90B), liver samples (10 samples per group) were collected and stored at −20 °C. The HSP90 concentration in liver tissues was detected using an enzyme-linked immunoassay [48].

### 2.9. Histological Examination

Two-centimeter samples from the spleen, duodenum, jejunum, and ileum were separated, which were then preserved in 10% neutral buffered formaldehyde (NBF) for 72 h before being dehydrated, cleaned, and placed in wax. The histological examination was carried out on 5-μm dense oblique sections (cut with a microtome). The sections were placed on slides and stained with hematoxylin and eosin (H&E) [49]. A computer-assisted digital-image pro plus (IPP) analysis program (Image-Pro Plus 4.5, Media Cybernetics, Silver Spring, MD, USA) and an OLYMPUS TH4-200 camera were used to assess the intestinal morphometry (villous height (VH), villous width (VW), crypt depth (CD), and muscular coat thickness (MCT)).

### 2.10. Immunohistochemical Procedures

The avidin-biotin-peroxidase complex (ABC) method was used for immunohistochemical labeling of CD3 and CD20 [50]. To put it briefly, intestinal sections encased in paraffin were deparaffinized using xylene and then rehydrated in ethyl alcohol. Tissue slices were treated with endogenous peroxidase blocking reagent (DAKO peroxidase blocking reagent, Cat. No. S 2001), a mixture of hydrogen peroxide and sodium azide. Two drops of the supersensitive mouse anti-Chicken CD3, clone CT-3 (Bio-Rad Lab., Dubai, UAE), and CD20 (ThermoFisher Scientific, Waltham, MA, USA) were applied to the sections. The sections were counterstained with hematoxylin, and the slides were examined under a microscope. According to Rizzardi, et al. [51], ImageJ software (IJ 1.46r, 2012, National institutes of health NIH, WA, USA) was used to determine the percentages of immunostaining-positive regions in five sections from each group at high magnification (×200).

### 2.11. Statistical Analysis

Data were analyzed with a one-way analysis of variance (ANOVA) using the GLM procedure in SPSS (Version 16 for Windows, SPSS Inc., Chicago, IL, USA) after the Shapiro–Wilk test was used to verify the normality, and Levene’s test was used to verify homogeneity of variance components among experimental treatments. The pen served as the experimental unit for growth performance measures, while individual birds were the experimental units for other parameters. Tukey’s test compared the differences between the means at a 5% probability level. Data variance was expressed as pooled SEM, and the significance level was set at *p* < 0.05.

## 3. Results

### 3.1. Growth Performance

During the starter and grower period, the HA group showed higher BW and BWG and improved FCR compared with other groups (*p* < 0.001). The SP group and SP+HA group showed improved growth performance compared with the POS CON group. The FI was lower in the SP group during the starter period and in the SP and SP+HA groups in the grower period compared with the NEG CON (*p* < 0.001). During the finisher period, the BW was higher in the HA group compared to other experimental groups (*p* < 0.001), while the BWG was similar to the NEG CON (*p* < 0.001). The SP, HA, and SP+HA groups showed improved growth performance compared to the POS CON. The NEG CON showed higher FI than all other groups (*p* < 0.001). The overall performance was improved in the experimental groups in this order: HA > NEG CON > SP > SP+HA > POS CON (*p* < 0.001) (Table 3). During the acute cyclic heat stress period, the addition of HA resulted in the highest growth performance among all experimental groups. The SP and the SP+HA groups also showed better performance than the POS CON group.

### 3.2. Meat Quality

As shown in Table 4, the pH of breast meat did not differ among the experimental groups (*p* > 0.05). The sensory characteristics of the breast muscles (color, odor, and consistency) were improved in the SP, HA, and SP+HA compared with the POS CON (*p* < 0.001), but they did not differ from the NEG CON. The cooking and thawing losses were lower in the SP, HA, and SP+HA groups compared with the NEG CON and POS CON (*p* < 0.001). The WHC was higher in the SP group and HA compared with other groups (*p* < 0.001).

### 3.3. Proximate Chemical Composition and Fatty Acid Composition of Breast Muscles

The moisture content of the breast meat was higher in the HA and SP+HA groups compared with the NEG CON (*p* = 0.007). The crude protein content was higher in the SP group and lower in the SP+HA group compared with the NEG CON (*p* < 0.001). The ash content was higher in the HA group compared with other experimental groups (*p* < 0.001). The fat content was not significantly different among the experimental groups (*p* > 0.05) (Table 5). The fatty acid composition of the breast muscles did not differ significantly among the experimental groups (*p* > 0.05) (Table 6).

### 3.4. Hematology and Phagocytosis

The RBC count and Hb level were lower in the POS CON compared with other groups (*p* < 0.05). The PCV%, MCV, MCH, and MCHC% were not significantly changed among the experimental groups (*p* > 0.05). The platelet count was lower in the HA group compared with other groups (*p* = 0.011). The WBCs, eosinophil, and basophil counts were not significantly changed among the experimental groups (*p* > 0.05). The lymphocyte and monocyte count were lower, while the neutrophil count was higher in the POS CON compared with the NEG CON (*p* < 0.05). The phagocytic % and phagocytic index were higher in the SP group than in the NEG and POS CON. They were higher in the HA and SP+HA groups than the POS CON (*p* < 0.01) (Table 7).

### 3.5. Thyroid Hormones, Immune Status, and Heat Shock Proteins

The serum levels of T3 and T4 were higher in the HA and SP+HA groups compared with the NEG CON and POS CON groups (*p* < 0.01). The serum concentrations of IL-10, C3, and lysozymes were higher in the SP, HA, and SP+HA groups compared with the NEG and POS CON, and the highest concentrations were recorded in the SP+HA group (*p* < 0.001). The liver concentrations of HSP90A and HSP90B were higher in the SP, HA, and SP+HA groups compared with the NEG and POS CON, and the highest concentrations were recorded in the SP+HA group (*p* < 0.001) (Table 8).

### 3.6. Spleen Histomorphology

**Figure 1:**
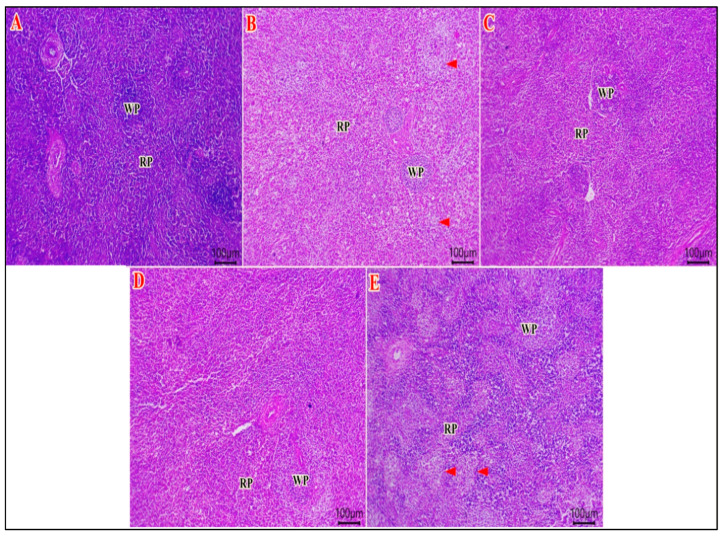
Photomicrograph of H&E-stained sections of the spleen. (**A**) The NEG CON showed normal histology of white pulp lymphoid follicles around eccentric arterioles and normal red pulp. (**B**) The POS CON showed depletion at most lymphoid populations of white pulp (arrowheads) and preserved red pulp. (**C**,**D**) The SP and HA groups showed apparent normal histological structures of white pulp & red pulp with a few numbers of necrotic or apoptotic lymphoid elements. (**E**) The SP+HA group showed intense white pulp (arrowheads) depletion and dilated red pulps: white pulp (WP) and red pulp (RP). Scale bar 100 μm.

The spleen tissues of the NEG CON group showed normal histology of white pulp lymphoid follicles around eccentric arterioles and normal red pulp. The latter had sinusoids filled with erythrocytes, lymphocytes, reticular fibers, and many macrophages. Meanwhile, the spleen tissues of the POS CON revealed depletion of most lymphoid populations of white pulp and preserved red pulp. The spleen tissues of the SP and HA groups revealed apparent normal histological structures of white pulp and red pulp with a few necrotic or apoptotic lymphoid elements. The spleen tissue of the SP+HA displayed intense depletion of white pulps beside dilated red pulps (Figure 1).

### 3.7. Intestinal Histomorphology

**Figure 2:**
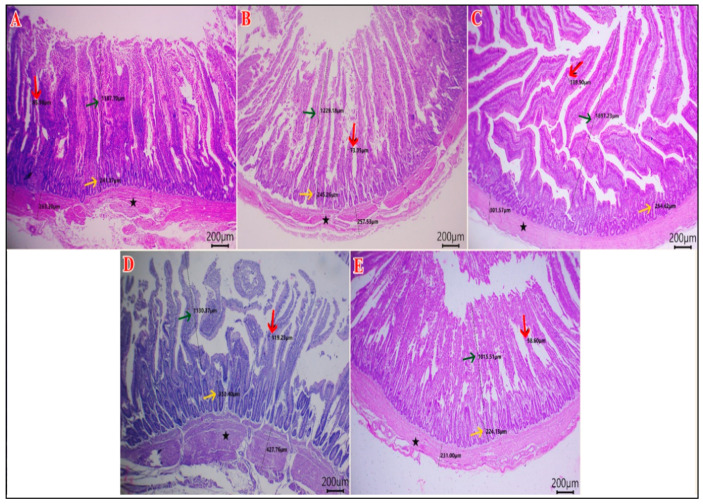
Photomicrograph of H&E-stained sections of duodenum showing: VH “villous height” (green arrows), VW “villous width “(red arrow), and CD “crypt depth” (yellow arrows), and MCT “muscular coat thickness” (black star). (**A**) NEG CON, (**B**) POS CON, (**C**) SP, (**D**) HA, (**E**) SP+HA. Scale bar 200 μm.

**Figure 3:**
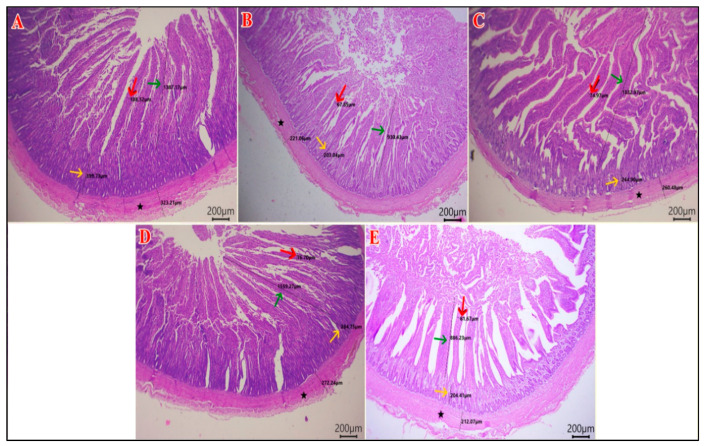
Photomicrograph of H&E-stained sections of jejunum showing: VH “villous height” (green arrows), VW “villous width “(red arrow), and CD “crypt depth” (yellow arrows), and MCT “muscular coat thickness” (black star). (**A**) NEG CON, (**B**) POS CON, (**C**) SP, (**D**) HA, (**E**) SP+HA. Scale bar 200 μm.

**Figure 4:**
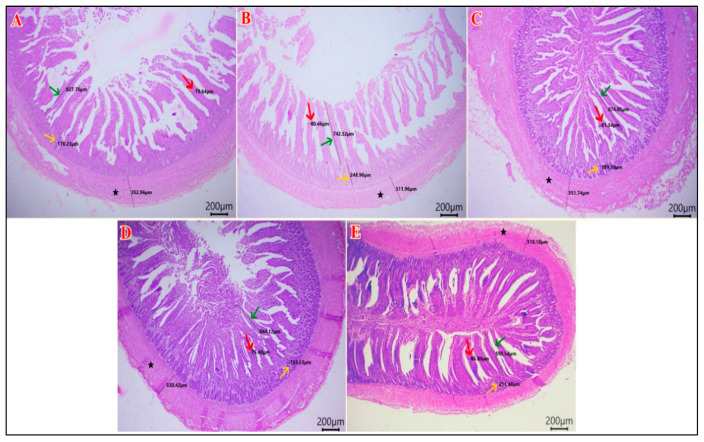
Photomicrograph of H&E-stained sections of ileum showing: VH “villous height” (green arrows), VW “villous width “(red arrow), and CD “crypt depth” (yellow arrows), and MCT “muscular coat thickness” (black star). (**A**) NEG CON, (**B**) POS CON, (**C**) SP, (**D**) HA, (**E**) SP+HA. Scale bar 200 μm.

As shown in Table 9, the duodenal villous height (VH) and width (VW) were greater in the HA group compared to the other groups (*p* < 0.01). The duodenal crypt depth (CD) was greater in the SP and the HA groups (*p* = 0.035). The highest VH: CD was in the HA group and the NEG CON, and the lowest value was in the SP group (*p* = 0.011). The muscular coat thickness (MCT) of the duodenum was the highest in the SP and HA groups (*p* < 0.001) (Figure 2). The jejunal VH was higher in the SP and HA groups compared with the POS CON (*p* < 0.001). The jejunal CD was lower in the POS CON and the SP+HA group compared with the NEG CON (*p* = 0.003). The VH: CD was higher in the SP and HA groups compared with the other groups (*p* < 0.001). The jejunal VW and MCT values did not differ among the experimental groups (*p* > 0.05) (Figure 3). The highest ileal VH and MCT values were observed in the HA group compared with other groups (*p* < 0.05). The VH: CD was higher in the SP and HA groups compared with the POS CON (*p* < 0.001). The ileal VW and CD did not differ among the experimental groups (*p* > 0.05) (Figure 4).

### 3.8. Immunohistochemistry

**Figure 5:**
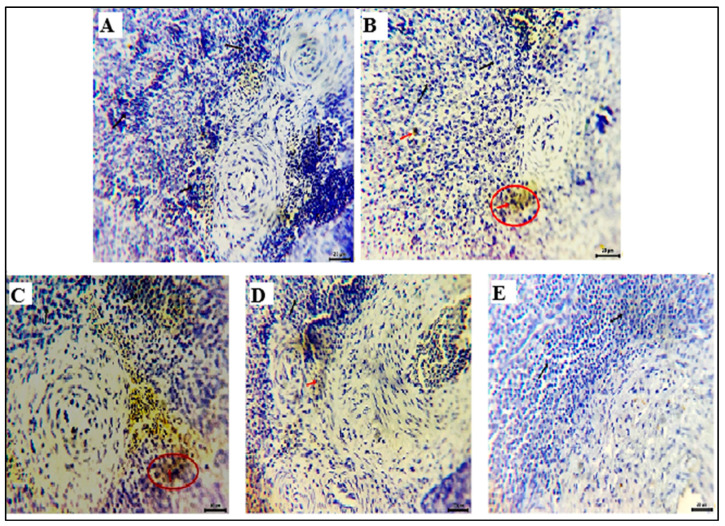
Photomicrographs from chicken’s spleen immunostained with monoclonal antibody against CD3+ T cells surface antigen, showing the % of the expressed antigen as brown cytoplasmic staining reaction of moderate intensity (red arrows). Black arrows point to negative cells. (**A**) NEG CON, (**B**) POS CON, (**C**) SP, (**D**) HA, (**E**) SP+HA. Scale bars 20 μm.

**Figure 6:**
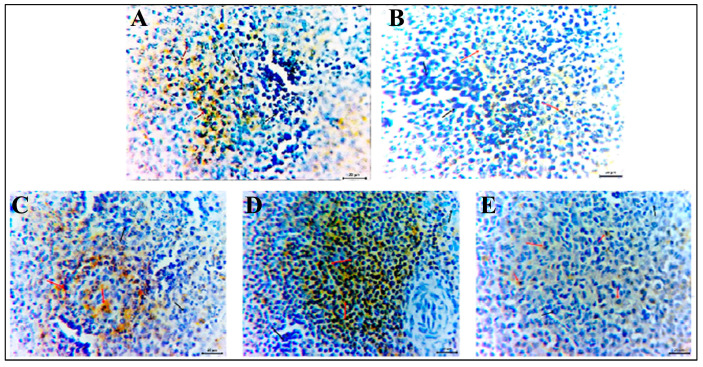
Photomicrographs from chicken’s spleen immunostained by monoclonal antibody against CD20+ B cells surface antigen, showing the % of the expressed antigen as brown cytoplasmic staining reaction of moderate intensity (red arrows). Black arrows point to negative cells. (**A**) NEG CON, (**B**) POS CON, (**C**) SP, (**D**) HA, (**E**) SP+HA. Scale bars 20 μm.

Examined sections from chicken’s spleen, immune-stained by specific monoclonal antibodies against CD3+ T lymphocytes surface receptor antigen, demonstrate 2.11, 0.28, zero, 2.77, and zero % positivity to the used marker in the NEG CON, POS CON, SP, HA, and SP+HA groups, respectively. The cellular cytoplasm exhibited moderate staining intensities (Figure 5A–E). Spleen sections immune-stained against CD20+ B lymphocytes showed 27.10, 23.70, 38.55, 67.38, and 13.23% in the corresponding groups with a moderate cytoplasmic staining reaction (Figure 6A–E).

## 4. Discussion

High ambient temperatures are one of the most common environmental stressors that have a detrimental impact on the welfare, health, and productivity of commercial poultry. The current study evaluated the role of single and combined dietary addition of *Spirulina platensis* (SP) and humic acid (HA) in mitigating the effects of acute cyclic heat stress on growth performance, meat quality, immune status, and intestinal morphology in broiler chickens. The results showed that HA addition improved the growth performance parameters than other groups during the starter period (no heat stress). During the grower and finisher periods (periods of acute cyclic heat stress), growth parameters (BW, BWG, and FCR) improved by the addition of HA, SP, and their combination compared to the POS CON group. However, the single addition of HA or SP yielded better growth performance than their combination, and the HA group provided the best results. The mechanism underlying the poor SP+HA performance remains unclear. However, one plausible explanation may be that HA, due to its strong chelating and adsorptive properties, may bind nutrients, trace elements, or bioactive molecules from spirulina, thereby reducing their bioavailability and utilization [52]. Since no direct measurements support the suggested mechanism, further studies are necessary to elucidate the biological interactions between the additives under heat-stress conditions. Heat stress impairs nutrient utilization and digestibility, which has a discernible negative impact on output performance. It causes a considerable decrease in the activity of the enzymes involved in protein digestion [53]. It was proposed that stressed birds expend greater energy to adapt to environmental stressors while allocating less energy towards growth. This may be partly responsible for the poor growth performance of heat-stressed birds [54]. In contrast, the higher growth performance of birds fed dietary additives may be due to their beneficial effects on feed utilization and nutrient absorption [55,56]. In addition, the improved growth in the SP and HA groups in the present study may be attributed to the reported improvements in gut histomorphology and the possible modulation of immune cell profiles, which together may have contributed to reduced physiological stress. Khosravinia [57] found that adding phytogenic compounds to the drinking water of heat-stressed broiler chickens encourages the digestive process and produces a modest improvement in FCR, and consequently increases poultry production efficiency. Prior research demonstrated that HA improved broiler chicken production performance [58,59]. Ozturk and Coskun [60] showed that humic acid (1.7 ppm) increased live weight gain without negatively affecting FCR. The addition of HA in broiler diets improved nutrient absorption and growth performance by enhancing gut health [61]. Moreover, humic acid’s chelating and membrane-stabilizing properties limit oxidative damage, improve nutrient digestibility, increase mineral bioavailability, and modify gut microbiota [62,63].

Spirulina has been utilized as a dietary and functional feed component due to its high nutritional content and valuable properties [55,64]. Additionally, the protein found in SP provides a well-balanced and highly digestible amino acid profile [65]. The beneficial effects of SP on the growth performance of heat-stressed broiler chicks may be related to the physiological roles of bioactive chemicals found in SP, such as phenolic compounds, carotenoids, vitamins, minerals, γ-linolenic acid, and essential amino acids [66]. According to Kharde et al. [67], broiler chickens fed a diet supplemented with 300 or 500 mg/kg of spirulina for six weeks showed a substantial increase in mean body weight, weight gain, and feed efficiency compared to the control group. Abdel-Moneim, et al. [68] demonstrated that 5–10 g SP enhanced the growth performance, carcass yield, antioxidant status, and immunological function of broiler chickens raised under heat stress conditions and fed a basal diet of corn and soybean meal.

Regarding the composition and the quality of the breast muscles, the current study showed that the crude protein content of the breast muscles was higher in the SP group and lower in the SP+HA group compared with the NEG CON. These results can explain the growth outcomes. The higher crude protein content in the SP group can be attributed to spirulina’s high metabolizable energy content [65] and a complete protein profile, which includes all essential amino acids [55]. However, the addition had no significant effect on the fatty acid composition of the breast muscles. Additionally, the pH of breast muscles remains unchanged. While the SP or HA addition resulted in better odor, color, and consistency than the POS CON, and the SP group showed better scores. An improvement in meat consistency was noted in the SP group, followed by the SP+HA group.

Factors such as WHC, drip loss, and cooking loss are crucial in assessing meat quality, as water loss during dripping can easily lead to the loss of many nutrients [69]. Water-holding capacity is one of the essential attributes of meat quality that affects its freshness. Meat with a higher WHC has a reduced drip and cooking loss, indicating a relationship between the two [70]. In the current study, both cooking and thawing losses were lower in the SP and HA groups when used individually, which was more effective than their mixture. In addition, the WHC was higher in the SP, HA, and SP+HA groups compared to the control groups. According to Deng, et al. [71], noticeable protein denaturation occurred due to the elevated metabolic rate during heat stress. Zhang, et al. [72] found that the breast muscles of heat-stressed chickens experienced higher cooking loss due to more severe protein denaturation, which reduced the protein’s capacity to bind to water. El-Bahr, et al. [73] found that drip loss was considerably reduced when broiler diets containing 0.1% spirulina were used. Park et al. [56] demonstrated that adding 0.25–1% Spirulina to broiler diets significantly decreased drip loss after seven days of storage. The delayed oxidation of the cell membrane induced by SP’s antioxidant components, such as phycocyanin and phenolic compounds, is the reason for these outcomes, as SP contains 70% antioxidant activity, which can reduce oxidation.

Hematological indices assess the extent of stress induced by various stressors and are considered reliable indicators of an animal’s health [74]. The current study showed lower RBC count, Hb level, lymphocyte, and monocyte counts in the POS CON. The platelet count was higher in the HA group, while the neutrophil count was greater in the POS CON. These results indicate that SP and HA individually improved the hematological parameters in broilers exposed to acute cyclic heat stress, whereas their combination produced less consistent effects. In addition to carrying oxygen, avian erythrocytes also play an active role in immune responses by producing cytokine-like molecules, upregulating genes involved in viral and bacterial responses, and sequestering pathogens through phagocytosis or surface binding [75].

Hemoglobin (Hb), red blood cells (RBCs), and packed cell volume (PCV) have all been shown to increase when dietary HA is added [76,77]. This may be because HA affects binding inorganic ions and delivers nutrients to cells [61]. The beneficial effects observed in birds receiving humic acid could be partly explained by its antioxidant potential, described in earlier studies, which showed enhanced activities of glutathione reductase and catalase and reduced lipid peroxidation markers [62,78]. The immunostimulatory properties of humic acid were studied to enhance immune function and improve the overall health of animals. In both humans and animals, some spirulina species, such as *Spirulina platensis*, have demonstrated immunomodulatory qualities [79]. Kolluri et al. [80] showed that drinking water containing SP at concentrations of 10, 15, and 20 g/L significantly increased the hemoglobin levels of heat-stressed broiler chickens compared to their control counterparts. They suggested that the increased hemoglobin concentration in treated birds may be due to the higher iron content of SP [81].

Poultry thermoregulation is a complicated process that involves immunological, hormonal, and neurological processes. Blood biochemical measurements serve as essential indicators for examining these reactions and calculating the heat tolerance of broiler chickens. Yahav [82] found that low thyroid hormone levels in heat-stressed chickens are caused by decreased glandular activity, which is inversely correlated with ambient temperature and is associated with a reduced metabolic rate. In the current study, the increase in T3 and T4 levels observed in the HA and SP+HA groups may reflect an adaptive metabolic adjustment to heat stress rather than a direct improvement in thermotolerance. Further evidence is needed to confirm whether these hormonal changes represent enhanced resilience or a transient hypermetabolic response. El-kelawy et al. [62] reported increased levels of thyroid hormones in broiler chickens’ diets containing HA at levels of 1 and 2 g/kg diet. Abed, et al. [34] reported increased T3 and T4 levels in heat-stressed broiler chickens by adding SP at 1–2 g/kg diet.

In response to HS, animals increase their production of HSPs as a protective mechanism against cellular damage. Under normal physiological conditions, heat shock proteins (HSPs) are maintained at low basal concentrations. However, exposure to external stressors, such as high temperatures, causes their expression to increase significantly, which helps mitigate cell damage caused by the stress [83]. In the present study, liver concentrations of HSP90A and HSP90B were higher in the SP, HA, and SP+HA groups compared with both controls, with the highest levels observed in the SP+HA group. This upregulation reflects an activated cellular defense mechanism rather than a direct improvement in thermotolerance. Increased HSP90 expression indicates that birds receiving SP and HA mounted a stronger protective response to heat-induced cellular stress, supporting protein stabilization and refolding processes. However, elevated HSP levels generally reflect increased cellular stress load. Therefore, additional evidence would be required to determine whether this response represents enhanced resilience or simply greater activation of stress-protection pathways. HSPs offer protection by maintaining the stability of cytoskeletal and other cellular proteins, particularly in scenarios of oxidative stress, where denaturation may be caused by reactive oxygen species (ROS) [84]. Heat shock proteins function as molecular chaperones that detect cellular redox alterations and associate with unfolded and misfolded proteins to facilitate their restoration to a functional conformation [85]. Broiler HSP90 levels increase in several tissues within two hours of HS [86]. Heat stress can double HSP90 levels, which promotes cellular homeostasis and a productive stress response [87,88].

One of the most apparent consequences of HS in broilers is immunosuppression, which is defined by changes in humoral and cell-mediated immunity [89,90]. Extracellular signaling peptides known as cytokines can modulate the immune system. Immunosuppression may result from downregulated cytokine expression during HS [91]. Immune responses depend critically on the balance between proinflammatory and anti-inflammatory cytokines. A key anti-inflammatory cytokine that regulates the immune response in various pathological circumstances is interleukin-10 [92,93]. Conversely, the complement system, including C3, is a crucial part of the innate immune response that, when activated, leads to inflammation to help fight off invaders and clear damaged cells [94]. Lysozymes are known to help eradicate intestinal infections in chickens through various mechanisms, such as enhancing the phagocytic activity of macrophages [95]. Different cell surface markers are used to identify different lymphocyte subpopulations. The T-cell co-receptor CD3 initiates a signaling cascade that activates helper and cytotoxic T cells in response to antigen recognition [96]. B-cell activity, differentiation, and proliferation are all influenced by the B-lymphocyte surface antigen, CD20 [97]. Heat stress induces oxidative and inflammatory stress [98,99], which reduces the initiation of specific immunological responses by impairing the capacity of antigen-presenting cells to activate T cells [100].

In the current study, the serum concentrations of IL-10, complement 3, and lysozymes were higher in the SP, HA, and SP+HA groups, and the highest concentrations were recorded in the SP+HA group. The study also showed an improvement in phagocytic % and phagocytic index with the addition of SP, HA, and their combination compared with the POS CON, with the best results observed in the SP group. These findings showed that the dietary addition of SP, HA, or their combination enhanced innate immunity. Moreover, the HA group showed up-regulation in the immune expression of CD3 and CD20 proteins in the spleen tissues. In addition, the spleen morphology in the current study was better in the SP and HA groups compared with the POS CON, which was severely affected by acute cyclic heat stress. According to our findings thus far, the single addition of SP and HA offers a comprehensive immunomodulatory impact that may mitigate the adverse effects of acute cyclic HS in poultry by promoting both humoral and cell-mediated immunity. In the present study, the combination of SP and HA improved growth performance compared with the heat-stressed positive control, but it did not match the benefits observed with each additive used alone. Birds in the SP+HA group showed reduced breast muscle crude protein content, less favorable intestinal morphology, and more pronounced splenic lymphoid depletion than those in the SP or HA groups. At the same time, the combined treatment elicited the highest serum levels of IL-10, complement 3, and lysozyme, as well as the greatest hepatic concentrations of HSP90A and HSP90B. This pattern suggests that SP+HA induced a stronger activation of innate immune and cellular stress-response pathways without a proportional improvement in growth or tissue integrity, thereby potentially increasing the physiological cost of coping with heat stress. The mechanisms underlying this apparent lack of additivity remain unclear. They may involve altered nutrient utilization, interactions between spirulina bioactives and humic substances, or an excessive stimulation of stress- and immune-related pathways. These hypotheses, however, are speculative and warrant further targeted investigation. The bioactive chemicals found in SP, including phycocyanin, carotenoids, γ-linolenic acid, and essential amino acids, improve lipid metabolism, immunological response, and antioxidant defense in the face of heat or oxidative stress [68,101]. Furthermore, the crude polysaccharides (α-glucans) derived from SP can markedly enhance the phagocytic capacity of macrophages, indicating potential immunomodulatory activity [102]. Humic acid limits oxidative damage and modifies the gut microbiota [62,63]. Higher amounts of flavonoids and sulfalipids may cause cytokine production, which in turn may mediate immunological regulation of SP [103]. Zeweil et al. [104] demonstrated that SP can mitigate the adverse effects of long-term heat stress on the immune and growth performance of the local Gimmizah strain of chicken. The immune state can be enhanced by adding HA (up to 0.1%), primarily in low-nutrient-density, antibiotic-free diets [58]. Because HA is essential for the development of immune organs, particularly the thymus and bursa of Fabricius, its inclusion in the diet can enhance immunological development in broiler chickens [105]. According to El-Ratel, et al. [106], sodium humate (0.2–0.6%) improved the immune response of heat-stressed laying quail, as indicated by an increase in serum IgG and IgM. Cetin et al. [107] observed that adding humic acid to the diets of laying hens increased the number of lymphocytes by upregulating IL-2 receptors.

The functional capacity of the small intestine, which is crucial for nutrient absorption and digestion, is frequently evaluated using morphometric measures such as muscle thickness, VW, CD, and VH [108]. An improved absorptive surface area is typically associated with shallower CD, thicker muscles, and higher VH and VW [109]. Wider and longer villi increase the intestinal surface area, which is crucial for improving broiler chicken growth performance and maximizing nutrition absorption. Furthermore, an enhanced intestinal surface area may indicate increased intestinal bulk and length, further strengthening the capacity for nutrient absorption and utilization [110]. The current study demonstrated an improvement in various intestinal morphometric measures following the addition of HA. The jejunal and ileal VH: CD ratio was higher in the HA and SP groups compared with the POS CON group. While the combination of SP and HA did not show an improvement in the intestinal morphometry compared to the POS CON, this may also explain the lower growth performance of birds in the combined addition compared with the single addition of SP and HA. Both additives improve the intestinal morphology and integrity when used individually [111,112]. When SP is combined with HA’s chelating and acidic properties, these could irritate the intestinal mucosa or alter pH, causing villus atrophy or reduced villus height/crypt depth ratio. Under heat stress conditions, these negative interactions may be further exacerbated, as the intestinal barrier and antioxidant defense systems are already compromised [113].

The limitations of this study include that the acute cyclic heat stress experiment focused on a few hours and days, potentially failing to account for the cumulative impact of repeated heat waves or chronic stress. Further studies are warranted to clarify the biochemical interactions between SP and HA under different stress intensities. In addition, only one level of SP and HA was evaluated. The effect of graded levels of SP and HA on mitigating acute and chronic heat stress in broiler chickens should be evaluated in future research. Additionally, the inclusion of oxytetracycline before treatment allocation may have altered the gut microbiota and immune parameters.

## 5. Conclusions

According to the present study, dietary addition of HA or SP individually improved growth performance in heat-stressed broilers, with HA producing the most pronounced benefits. The combined addition did not provide additive advantages and, in several outcomes, was less effective than the individual additives. Dietary SP and HA each improved selected meat quality traits and activated innate immune and cellular-stress response pathways, as reflected by changes in IL-10, complement 3, lysozyme, and hepatic HSP90 isoforms. Overall, SP or HA used alone appeared more effective than their combination; however, the evidence does not confirm the mitigation of heat stress itself, but rather indicates enhanced activation of physiological defense mechanisms. Further studies are needed to clarify the biological interactions between the additives under heat-stress conditions.

## Figures and Tables

**Table 1:** Temperature, relative humidity, and temperature-humidity index.

Days	Minimum Temperature (°C)	Maximum Temperature (°C)	Relative Humidity (%)	Minimum THI	Maximum THI
1st	35.3	37.5	69	39.2	41.0
2nd	35.5	37.3	71	39.1	40.6
3rd	36.2	37.5	68	40.0	41.1
4th	35.0	36.5	70	38.8	40.0

THI: temperature-humidity index.

**Table 2:** Proximate chemical composition of the diets (%).

Items	Diet A ^1^			Diet B ^2^		
	Starter	Grower	Finisher	Starter	Grower	Finisher
Ingredients (%)						
Yellow corn	55.725	59.25	62.2	55.725	59.25	62.1
Soybean meal, 48%	33.53	28	23.6	33.33	28	23.5
Corn gluten, 60%	4	5.325	6	4	5.125	6
Spirulina, 63%	-	-	-	0.2	0.2	0.2
Soybean oil	2.2	3.1	4.095	2.2	3.1	4.095
Calcium carbonate	1.2	1.2	1.1	1.2	1.2	1.1
Dicalcium phosphate 18%	1.5	1.4	1.3	1.5	1.4	1.3
Common salt	0.15	0.15	0.15	0.15	0.15	0.15
Premix ^3^	0.3	0.3	0.3	0.3	0.3	0.3
DL-Methionine, 98%	0.4	0.3	0.33	0.4	0.3	0.33
Lysine, HCl 78%	0.47	0.45	0.4	0.47	0.45	0.4
Choline	0.07	0.07	0.07	0.07	0.07	0.07
Threonine	0.1	0.1	0.1	0.1	0.1	0.1
Phytase ^4^	0.005	0.005	0.005	0.005	0.005	0.005
Sodium bicarbonate	0.25	0.25	0.25	0.25	0.25	0.25
Antimycotoxin	0.1	0.1	0.1	0.1	0.1	0.1
Chemical composition (%)						
DM	90.25	90.4	90.5	90.3	90.45	90.6
ME (kcal/kg)	3003	3107	3208	3003	3105	3207
CP	23.12	21.50	20.02	23.15	21.51	20.09
Lysine	1.47	1.31	1.16	1.47	1.32	1.16
Methionine	0.72	0.61	0.63	0.72	0.61	0.63
Calcium	0.94	0.90	0.83	0.94	0.90	0.83
Av. P	0.48	0.45	0.42	0.48	0.45	0.42

^1^ Diet A for the NEG CON, POS CON, and HA-supplemented groups. ^2^ Diet B for the SP-added group and HA+SP-added group. DM: dry matter. CP: crude protein. ME: Metabolizable energy. Av. P: available phosphorus. ^3^ Vitamin and mineral premix per kg of diet: vitamin K3; 3.2 mg, vitamin E; 80 IU, vitamin A; 12,000 IU, vitamin D3; 5000 IU, thiamine; 3.2 mg, pantothenic acid; 20 mg, riboflavin; 8.6 mg, pyridoxine; 4.3 mg, folic acid; 2.2 mg, vitamin B12; 0.017 mg, niacin; 65 mg, biotin; 0.22 mg, Fe; 20 mg, Cu; 16 mg, choline; 1650 mg, Mn; 120 mg, Zn; 110 mg, Se; 0.30 mg, I; 1.25 mg. ^4^ Phytase, RONOZYME^®^, DSM-Firmenich, Kaiseraugst, Switzerland.

**Table 3:** Effect of *Spirulina platensis* and humic acid addition on the growth performance of heat-stressed broiler chickens.

Items	NEG CON	POS CON	SP	HA	SP+HA	SEM	*p*-Value
Initial BW (g)	103	101	100	101	101	0.439	0.252
Starter period (4–10 d)							
BW (g)	301 ^b^	297 ^b^	301 ^b^	313 ^a^	288 ^c^	1.220	<0.001
BWG (g)	197 ^b^	196 ^b^	201 ^b^	211 ^a^	187 ^c^	1.240	<0.001
FI (g)	337 ^a^	332 ^ab^	330 ^b^	332 ^ab^	332 ^ab^	0.740	<0.001
FCR	1.71 ^ab^	1.69 ^bc^	1.64 ^cd^	1.57 ^d^	1.77 ^a^	0.011	<0.001
Grower period (11–23 d)							
BW (g)	1322 ^b^	1064 ^e^	1250 ^c^	1412 ^a^	1159 ^d^	17.10	<0.001
BWG (g)	1021 ^b^	767 ^e^	949 ^c^	1100 ^a^	871 ^d^	16.20	<0.001
FI (g)	1395 ^a^	1395 ^a^	1375 ^b^	1380 ^ab^	1373 ^b^	2.280	<0.001
FCR	1.37 ^d^	1.82 ^a^	1.45 ^c^	1.26 ^e^	1.58 ^b^	0.027	<0.001
Finisher period (24–35 d)							
BW (g)	2392 ^b^	1951 ^e^	2243 ^c^	2483 ^a^	2079 ^d^	28.00	<0.001
BWG (g)	1070 ^a^	887 ^c^	993 ^ab^	1071 ^a^	920 ^bc^	12.80	<0.001
FI (g)	1710 ^a^	1592 ^ab^	1521 ^b^	1541 ^b^	1518 ^b^	16.10	<0.001
FCR	1.60 ^bc^	1.80 ^a^	1.54 ^bc^	1.44 ^c^	1.65 ^ab^	0.024	<0.001
Overall performance							<0.001
BW (g)	2392 ^b^	1951 ^e^	2243 ^c^	2483 ^a^	2079 ^d^	28.00	<0.001
BWG (g)	2289 ^b^	1850 ^e^	2143 ^c^	2382 ^a^	1978 ^d^	27.90	<0.001
FI (g)	3442 ^a^	3318 ^ab^	3226 ^b^	3253 ^b^	3223 ^b^	17.20	<0.001
FCR	1.51 ^c^	1.79 ^a^	1.51 ^c^	1.37 ^d^	1.63 ^b^	0.021	<0.001

Variation in the data was expressed as pooled SEM (*n* = 100). ^a,b,c,d,e^ Means within the same row carrying different superscripts significantly differ at (*p* < 0.05). BW: body weight, BWG: body weight gain, FI: feed intake, FCR: feed conversion ratio. NEG CON: negative control group, the chicks received a basal diet and were maintained in thermoneutral conditions. POS CON: positive control group, the chicks received a basal diet and were exposed to acute cyclic heat stress. SP: chicks received a basal diet added with SP and were exposed to acute cyclic heat stress. HA: chicks received a basal diet added with HA and were exposed to acute cyclic heat stress. SP+HA: chicks received a basal diet added with SP and HA and were exposed to acute cyclic heat stress.

**Table 4:** Effect of *Spirulina platensis* and humic acid addition on the quality of the breast muscles (on 35 days).

Item	NEG CON	POS CON	SP	HA	SP+HA	SEM	*p*-Value
pH	5.85	6.01	5.87	6.00	5.92	0.030	0.350
Odor	4.16 ^a^	3.15 ^b^	4.13 ^a^	4.06 ^a^	4.05 ^a^	0.106	<0.001
Color	4.16 ^ab^	3.12 ^c^	4.15 ^a^	4.06 ^ab^	4.00 ^b^	0.107	<0.001
Consistency	4.00 ^ab^	3.30 ^c^	4.16 ^a^	3.86 ^b^	4.05 ^ab^	0.086	<0.001
Cooking loss	18.5 ^b^	18.8 ^a^	15.0 ^d^	15.7 ^c^	15.3 ^d^	0.440	<0.001
Thawing loss	5.32 ^b^	6.03 ^a^	4.73 ^c^	4.4 ^d^	4.53 ^d^	0.161	<0.001
WHC (%)	73.1 ^c^	71.2 ^d^	78.0 ^a^	78.0 ^a^	77.0 ^b^	4.750	<0.001

Variation in the data was expressed as pooled SEM (*n* = 10). ^a,b,c,d^ Means within the same row carrying different superscripts significantly differ at (*p* < 0.05). WHC: water holding capacity. NEG CON: negative control group, the chicks received a basal diet and were maintained in thermoneutral conditions. POS CON: positive control group, the chicks received a basal diet and were exposed to acute cyclic heat stress. SP: chicks received a basal diet added with SP and were exposed to acute cyclic heat stress. HA: chicks received a basal diet added with HA and were exposed to acute cyclic heat stress. SP+HA: chicks received a basal diet added with SP and HA and were exposed to acute cyclic heat stress.

**Table 5:** Effect of *Spirulina platensis* and humic acid addition on the proximate chemical composition of breast muscles of heat-stressed broiler chickens (on 35 days).

Item	NEG CON	POS CON	SP	HA	SP+HA	SEM	*p*-Value
Moisture %	70.5 ^b^	71.6 ^ab^	72.1 ^ab^	73.2 ^a^	73.3 ^a^	0.381	0.007
CP %	19.5 ^b^	20.5 ^ab^	21.3 ^a^	19.3 ^b^	17.3 ^c^	0.449	<0.001
Fat %	3.18	2.83	3.05	3.03	3.05	0.047	0.222
Ash%	0.665 ^c^	0.980 ^b^	0.640 ^c^	1.360 ^a^	0.855 ^bc^	0.087	<0.001

Variation in the data was expressed as pooled SEM (*n* = 10). ^a,b,c^ Means within the same row carrying different superscripts significantly differ at (*p* < 0.05). CP: crude protein. NEG CON: negative control group, the chicks received a basal diet and were maintained in thermoneutral conditions. POS CON: positive control group, the chicks received a basal diet and were exposed to acute cyclic heat stress. SP: chicks received a basal diet added with SP and were exposed to acute cyclic heat stress. HA: chicks received a basal diet added with HA and were exposed to acute cyclic heat stress. SP+HA: chicks received a basal diet added with SP and HA and were exposed to acute cyclic heat stress.

**Table 6:** Effect of *Spirulina platensis* and humic acid addition on fatty acid composition of the breast muscles of heat-stressed broiler chickens (on 35 days).

Items	NEG CON	POS CON	SP	HA	SP+HA	SEM	*p*-Value
C14:0	0.023	0.022	0.019	0.019	0.020	0.0006	0.089
C16:0	0.857	0.808	0.881	0.834	0.854	0.0102	0.212
C18:0	0.293	0.294	0.291	0.284	0.295	0.0016	0.227
C16:1	0.197	0.195	0.196	0.196	0.196	0.0011	0.889
C18:1	1.641	1.643	1.650	1.645	1.663	0.0038	0.544
C20:1	0.065	0.064	0.064	0.064	0.065	0.0013	0.969
C18:2	0.982	0.979	0.986	0.985	0.984	0.0016	0.400
C18:3	0.068	0.067	0.067	0.067	0.068	0.0012	0.919

Variation in the data was expressed as pooled SEM (*n* = 10). Fatty acid composition, expressed as a percentage of total fatty acids. NEG CON: negative control group, the chicks received a basal diet and were maintained in thermoneutral conditions. POS CON: positive control group, the chicks received a basal diet and were exposed to acute cyclic heat stress. SP: chicks received a basal diet added with SP and were exposed to acute cyclic heat stress. HA: chicks received a basal diet added with HA and were exposed to acute cyclic heat stress. SP+HA: chicks received a basal diet added with SP and HA and were exposed to acute cyclic heat stress.

**Table 7:** Effect of *Spirulina platensis* and humic acid addition on the erythrogram, leukogram, and phagocytosis in broiler chickens (on 35 days).

Items	NEG CON	POS CON	SP	HA	SP+HA	SEM	*p*-Value
Erythrogram							
RBCs (10^6^/cmm)	4.13 ^ab^	2.83 ^b^	3.65 ^ab^	3.87 ^ab^	4.32 ^a^	0.194	0.045
Hb (g/dL)	11.9 ^a^	9.04 ^b^	10.5 ^ab^	11.5 ^ab^	12.4 ^a^	0.423	0.024
PCV (%)	34.8	26.9	31.7	34.0	35.8	1.190	0.052
MCV (fl)	84.4	95.2	87.4	87.8	83.3	1.770	0.238
MCH (pg)	28.8	31.9	29.1	29.9	28.7	0.563	0.363
MCHC (%)	34.1	33.6	33.3	34.0	34.5	0.200	0.464
Platelets (10^3^/cmm)	155 ^b^	155 ^b^	166 ^b^	213 ^a^	191 ^ab^	8.040	0.011
Leukogram							
WBCs (10^3^/cmm)	19.8	17.1	18.7	18.6	19.0	0.339	0.104
Lymphocytes (10^3^/cmm)	14.5 ^a^	10.2 ^b^	13.3 ^a^	13.3 ^a^	13.4 ^a^	0.513	0.009
Neutrophils (10^3^/cmm)	3.55 ^b^	5.65 ^a^	3.86 ^b^	3.75 ^b^	3.91 ^b^	0.259	0.002
Monocytes (10^3^/cmm)	0.970 ^a^	0.690 ^b^	0.825 ^ab^	0.860 ^ab^	0.945 ^ab^	0.036	0.039
Eosinophils (10^3^/µ L)	0.475	0.450	0.435	0.500	0.475	0.021	0.934
Basophils (10^3^/cmm)	0.292	0.175	0.265	0.255	0.245	0.027	0.834
Phagocytosis							
Phagocytic %	56.5 ^bc^	49.0 ^c^	74.0 ^a^	61.0 ^bc^	66.0 ^ab^	2.910	0.003
Phagocytic index	2.33 ^b^	0.96 ^c^	3.25 ^a^	2.82 ^ab^	2.92 ^ab^	0.272	0.001

Variation in the data was expressed as pooled SEM (*n* = 10). ^a,b,c^ The estimated values in a single row with various superscripts differ considerably (*p* < 0.05). RBCs： red blood cells, Hb: hemoglobin, PCV: Packed cell volume, MCV: mean corpuscular volume, MCH: mean corpuscular hemoglobin, MCHC: mean corpuscular hemoglobin concentration, WBCs: white blood cells. NEG CON: negative control group, the chicks received a basal diet and were maintained in thermoneutral conditions. POS CON: positive control group, the chicks received a basal diet and were exposed to acute cyclic heat stress. SP: chicks received a basal diet added with SP and were exposed to acute cyclic heat stress. HA: chicks received a basal diet added with HA and were exposed to acute cyclic heat stress. SP+HA: chicks received a basal diet added with SP and HA and were exposed to acute cyclic heat stress.

**Table 8:** Effect of *Spirulina platensis* and humic acid addition on the thyroid hormones, immune status, and heat shock protein levels of heat-stressed broiler chickens (on 35 days).

Items	NEG CON	POS CON	SP	HA	SP+HA	SEM	*p*-Value
T3 (ng/mL)	2.41 ^b^	2.65 ^b^	3.27 ^ab^	3.66 ^a^	3.88 ^a^	0.168	<0.01
T4 (ng/mL)	18.7 ^b^	18.8 ^b^	20.6 ^b^	23.7 ^a^	25.7 ^a^	0.764	<0.001
IL10 (pg/mL)	1.33 ^d^	1.47 ^cd^	2.20 ^bc^	2.93 ^ab^	3.33 ^a^	0.219	<0.001
C3 (mg/dL)	1.07 ^c^	1.09 ^c^	1.19 ^b^	1.26 ^a^	1.31 ^a^	0.025	<0.001
Lysozymes (µg/mL)	128 ^c^	130 ^c^	136 ^bc^	141 ^b^	150 ^a^	2.210	<0.001
HSP90A (ng/mg tissue)	1.17 ^d^	1.50 ^cd^	1.63 ^c^	2.20 ^b^	2.83 ^a^	0.164	<0.001
HSP90B (ng/mg tissue)	0.40 ^b^	0.72 ^b^	1.37 ^a^	1.37 ^a^	1.53 ^a^	0.128	<0.001

Variation in the data was expressed as pooled SEM (*n* = 10). ^a,b,c,d^ Means within the same row carrying different superscripts significantly differ at (*p* < 0.05). T3: Triiodothyronine, T4: thyroxine, C3: complement 3, IL-10: interleukin-10. NEG CON: negative control group, the chicks received a basal diet and were maintained in thermoneutral conditions. POS CON: positive control group, the chicks received a basal diet and were exposed to acute cyclic heat stress. SP: chicks received a basal diet added with SP and were exposed to acute cyclic heat stress. HA: chicks received a basal diet added with HA and were exposed to acute cyclic heat stress. SP+HA: chicks received a basal diet added with SP and HA and were exposed to acute cyclic heat stress.

**Table 9:** Effect of *Spirulina platensis* and humic acid addition on the morphometric measures (μm) of the small intestine of heat-stressed broiler chickens (on 35 days).

Items	NEG CON	POS CON	SP	HA	SP+HA	SEM	*p*-Value
Duodenum							
VH	1253 ^ab^	1079 ^b^	1217 ^ab^	1615 ^a^	964 ^b^	69.0	0.007
VW	77.7 ^bc^	66.3 ^bc^	106 ^b^	159 ^a^	51.3 ^c^	10.7	<0.001
CD	242 ^ab^	225 ^b^	300 ^a^	299 ^a^	208 ^b^	13.0	0.035
VH: CD	5.17 ^a^	4.81 ^ab^	4.13 ^b^	5.37 ^a^	4.63 ^ab^	0.14	0.011
MCT	255 ^ab^	241 ^b^	390 ^a^	361 ^a^	209 ^b^	20.2	<0.001
Jejunum							
VH	1383 ^a^	858 ^b^	1595 ^a^	1457 ^a^	832 ^b^	90.2	<0.001
VW	199	81.3	92.7	92	81.3	21.0	0.363
CD	356 ^a^	233 ^b^	284 ^ab^	288 ^ab^	207 ^b^	15.7	0.003
VH:CD	3.92 ^b^	3.70 ^b^	5.59 ^a^	5.07 ^a^	4.03 ^b^	0.21	<0.001
MCT	332	243	288	295	249	11.8	0.072
Ileum							
VH	774 ^ab^	713 ^bc^	797 ^ab^	844 ^a^	651 ^c^	20.4	0.003
VW	80.7	78	74	81	72.7	1.92	0.594
CD	175	221	196	192	190	5.47	0.077
VH:CD	4.42 ^a^	3.25 ^b^	4.06 ^a^	4.41 ^a^	3.43 ^b^	0.14	<0.001
MCT	332 ^ab^	257 ^b^	327 ^ab^	351 ^a^	265 ^ab^	12.6	0.021

Variation in the data was expressed as pooled SEM (*n* = 10). ^a,b,c^ Means within the same row carrying different superscripts significantly differ at (*p* < 0.05). VH: villous height, VW: villous width, CD: crypt depth, MCT: muscular coat thickness. NEG CON: negative control group, the chicks received a basal diet and were maintained in thermoneutral conditions. POS CON: positive control group, the chicks received a basal diet and were exposed to acute cyclic heat stress. SP: chicks received a basal diet added with SP and were exposed to acute cyclic heat stress. HA: chicks received a basal diet added with HA and were exposed to acute cyclic heat stress. SP+HA: chicks received a basal diet added with SP and HA and were exposed to acute cyclic heat stress.

## Data Availability

The original contributions presented in this study are included in the article. Further inquiries can be directed to the corresponding authors.

## References

[1] Zhu L., Bao E., Zhao R., Hartung J. (2009). Expression of heat shock protein 60 in the tissues of transported piglets. Cell Stress Chaperones.

[2] Amer S.A., Gouda A., Hamed R.I., Nassar A.H., Ali H.S., Ibrahim R.M., Alagmy G.N., Abdelmoteleb A.M., Althobaiti F., Alotaibi K.S. (2025). The Effects of Fennel Essential Oil Supplementation on Mitigating the Heat Stress Impacts on Growth Rate, Blood Biochemical Parameters, and Liver Histopathology in Broiler Chickens. Vet. Sci..

[3] Gouda A., Amer S.A., Gabr S., Tolba S.A. (2020). Effect of dietary supplemental ascorbic acid and folic acid on the growth performance, redox status, and immune status of broiler chickens under heat stress. Trop. Anim. Health Prod..

[4] Temim S., Chagneau A.-M., Peresson R., Tesseraud S. (2000). Chronic heat exposure alters protein turnover of three different skeletal muscles in finishing broiler chickens fed 20 or 25% protein diets. J. Nutr..

[5] Olfati A., Mojtahedin A., Sadeghi T., Akbari M., Martínez-Pastor F. (2018). Comparison of growth performance and immune responses of broiler chicks reared under heat stress, cold stress and thermoneutral conditions. Span. J. Agric. Res..

[6] Aswathi P., Bhanja S., Kumar P., Shyamkumar T., Mehra M., Bhaisare D.B., Rath P.K. (2019). Effect of acute heat stress on the physiological and reproductive parameters of broiler breeder hens-A study under controlled thermal stress. Indian J. Anim. Res..

[7] Sugiharto S., Yudiarti T., Isroli I., Widiastuti E., Kusumanti E. (2017). Dietary supplementation of probiotics in poultry exposed to heat stress-a review. Ann. Anim. Sci..

[8] Vandana G., Sejian V., Lees A., Pragna P., Silpa M., Maloney S.K. (2021). Heat stress and poultry production: Impact and amelioration. Int. J. Biometeorol..

[9] Emery J. (2005). Heat Stress in Poultry-Solving the Problem. https://assets.publishing.service.gov.uk/government/uploads/system/uploads/attachment_data/file/69373/pb10543-heat-stress-050330.pdf.

[10] Mignon-Grasteau S., Moreri U., Narcy A., Rousseau X., Rodenburg T., Tixier-Boichard M., Zerjal T. (2015). Robustness to chronic heat stress in laying hens: A meta-analysis. Poult. Sci..

[11] Olanrewaju H., Purswell J., Collier S., Branton S. (2010). Effect of ambient temperature and light intensity on physiological reactions of heavy broiler chickens. Poult. Sci..

[12] Lin H., Zhang H., Jiao H., Zhao T., Sui S., Gu X., Zhang Z., Buyse J., Decuypere E. (2005). Thermoregulation responses of broiler chickens to humidity at different ambient temperatures. I. One week of age. Poult. Sci..

[13] Yahav S. (2000). Relative humidity at moderate ambient temperatures: Its effect on male broiler chickens and turkeys. Br. Poult. Sci..

[14] Abd El-Hack M.E., Abdelnour S.A., Taha A.E., Khafaga A.F., Arif M., Ayasan T., Swelum A.A., Abukhalil M.H., Alkahtani S., Aleya L. (2020). Herbs as thermoregulatory agents in poultry: An overview. Sci. Total Environ..

[15] Mirzaie S., Zirak-Khattab F., Hosseini S.A., Donyaei-Darian H. (2017). Effects of dietary Spirulina on antioxidant status, lipid profile, immune response and performance characteristics of broiler chickens reared under high ambient temperature. Asian-Australas. J. Anim. Sci..

[16] Khan M.I., Shin J.H., Kim J.D. (2018). The promising future of microalgae: Current status, challenges, and optimization of a sustainable and renewable industry for biofuels, feed, and other products. Microb. Cell Factories.

[17] Habib M.A.B., Parvin M., Huntington T.C., Hasan M.R. (2008). A Review on Culture, Production and Use of Spirulina as Food for Humans and Feeds for Domestic Animals.

[18] Kaushik P., Chauhan A. (2008). In vitro antibacterial activity of laboratory grown culture of Spirulina platensis. Indian J. Microbiol..

[19] Hirahashi T., Matsumoto M., Hazeki K., Saeki Y., Ui M., Seya T. (2002). Activation of the human innate immune system by Spirulina: Augmentation of interferon production and NK cytotoxicity by oral administration of hot water extract of *Spirulina platensis*. Int. Immunopharmacol..

[20] Hsiao G., Chou P.-H., Shen M.-Y., Chou D.-S., Lin C.-H., Sheu J.-R. (2005). C-phycocyanin, a very potent and novel platelet aggregation inhibitor from *Spirulina platensis*. J. Agric. Food Chem..

[21] Khan M., Shobha J.C., Mohan I.K., Naidu M.U.R., Sundaram C., Singh S., Kuppusamy P., Kutala V.K. (2005). Protective effect of Spirulina against doxorubicin-induced cardiotoxicity. Phytother. Res. Int. J. Devoted Pharmacol. Toxicol. Eval. Nat. Prod. Deriv..

[22] Khan M., Shobha J.C., Mohan I.K., Rao Naidu M.U., Prayag A., Kutala V.K. (2006). Spirulina attenuates cyclosporine-induced nephrotoxicity in rats. J. Appl. Toxicol. Int. J..

[23] Al-Qahtani W.H., Binobead M.A. (2019). Anti-inflammatory, antioxidant and antihepatotoxic effects of Spirulina platensis against D-galactosamine induced hepatotoxicity in rats. Saudi J. Biol. Sci..

[24] Nagaoka S., Shimizu K., Kaneko H., Shibayama F., Morikawa K., Kanamaru Y., Otsuka A., Hirahashi T., Kato T. (2005). A novel protein C-phycocyanin plays a crucial role in the hypocholesterolemic action of Spirulina platensis concentrate in rats. J. Nutr..

[25] Abd El-Hady A., El-Ghalid O. (2018). Spirulina platensis Algae (SPA): A novel poultry feed additive. Effect of SPA supplementation in broiler chicken diets on productive performance, lipid profile and calcium-phosphorus metabolism. Worlds Poult. Sci. J..

[26] Bonos E., Kasapidou E., Kargopoulos A., Karampampas A., Nikolakakis I., Christaki E., Florou-Paneri P. (2016). Spirulina as a functional ingredient in broiler chicken diets. S. Afr. J. Anim. Sci..

[27] Evans A., Smith D., Moritz J. (2015). Effects of algae incorporation into broiler starter diet formulations on nutrient digestibility and 3 to 21 d bird performance. J. Appl. Poult. Res..

[28] Yoruk M., Gul M., Hayırlı A., Macıt M. (2004). The effects of supplementation of humate and probiotic on egg production and quality parameters during the late laying period in hens. Poult. Sci..

[29] Islam K.M.S., Schumacher A., Gropp J. (2005). Humic acid substances in animal agriculture. Pak. J. Nutr..

[30] Rath N., Huff W., Huff G. (2006). Effects of humic acid on broiler chickens. Poult. Sci..

[31] Humin T. (2004). Humin animal feed supplements and veterinary medicine and humic acid based products. Humintech-Humintech GmbH Heerdter Landstr.

[32] Gomez-Rosales S., de L Angeles M. (2015). Addition of a worm leachate as source of humic substances in the drinking water of broiler chickens. Asian-Australas. J. Anim. Sci..

[33] El-shennawy E.-s., Zakkar A., Sulieman A.E., El-badawi A. (2025). Effect of Spirulina Powder on Quality and Sensory Attributes of Catfish Burger. Egypt. J. Chem..

[34] Abed H., Ahmed A., Abdelazeem F., Shourrap M. (2023). Effect of spirulina platensis algae supplementation on growth performance, physiological status of broilers during summer season. Egypt. J. Nutr. Feed..

[35] Hassan S.M. (2014). Effect of Adding Dietary Humate on Productive Performance of Broiler Chicks. Asian J. Poult. Sci..

[36] Aviagen Research (2014). Ross Broiler Management Manual, 2009.

[37] Jarraud M. (2008). Guide to Meteorological Instruments and Methods of Observation (WMO-No. 8).

[38] Association A.V.M. (2013). AVMA Guidelines for the Euthanasia of Animals.

[39] Petracci M., Baéza E. (2011). Harmonization of methodologies for the assessment of poultry meat quality features. World’s Poult. Sci. J..

[40] Castellini C., Mugnai C., Dal Bosco A. (2002). Effect of organic production system on broiler carcass and meat quality. Meat Sci..

[41] Belitz H.-D., Grosch W., Schieberle P. (2009). Meat. Food Chemistry.

[42] AOAC (2000). Official Methods of Analysis of AOAC International.

[43] Yang F., Liao J., Pei R., Yu W., Han Q., Li Y., Guo J., Hu L., Pan J., Tang Z. (2018). Autophagy attenuates copper-induced mitochondrial dysfunction by regulating oxidative stress in chicken hepatocytes. Chemosphere.

[44] Karsan A., Maclaren I., Conn D., Wadsworth L. (1993). An evaluation of hemoglobin determination using sodium lauryl sulfate. Am. J. Clin. Pathol..

[45] Hampton M.B., Vissers M.C., Winterbourn C.C. (1994). A single assay for measuring the rates of phagocytosis and bacterial killing by neutrophils. J. Leucoc. Biol..

[46] Omar A., Al-Khalaifah H., Mohamed W., Gharib H., Osman A., Al-Gabri N., Amer S. (2020). Effects of phenolic-rich onion (*Allium cepa* L.) extract on the growth performance, behavior, intestinal histology, amino acid digestibility, antioxidant activity, and the immune status of broiler chickens. Front. Vet. Sci..

[47] Lie Ø., Syed M., Solbu H. (1986). Forbedrede agarplatemetoder til bestemmelse av bovint lysozym og hemolytisk complement aktivitet. Acta Vet. Scand..

[48] Anderson R., Wang C., Van Kersen I., Lee K., Welch W., Lavagnini P., Hahn G. (1993). An immunoassay for heat shock protein 73/72: Use of the assay to correlate HSW3/72 levels in mammalian cells with heat response. Int. J. Hyperth..

[49] Suvarna S., Layton C., Bancroft J.D. (2013). Theory and Practice of Histological Techniques.

[50] Hsu S.M., Raine L., Fanger H. (1981). Use of avidin-biotin-peroxidase complex (ABC) in immunoperoxidase techniques: A comparison between ABC and unlabeled antibody (PAP) procedures. J. Histochem. Cytochem. Off. J. Histochem. Soc..

[51] Rizzardi A.E., Johnson A.T., Vogel R.I., Pambuccian S.E., Henriksen J., Skubitz A.P., Metzger G.J., Schmechel S.C. (2012). Quantitative comparison of immunohistochemical staining measured by digital image analysis versus pathologist visual scoring. Diagn. Pathol..

[52] Chen J., Yan C., Luo J., Fan L., Shi H., Gu H., Ji Y. (2025). Bioavailability and recalcitrance of humic-bonded dissolved organic nitrogen for algae uptake: Insights into eutrophication and metal interactions. Sci. Total Environ..

[53] Sahin K., Kucuk O., Sahin N., Sari M. (2002). Effects of vitamin C and vitamin E on lipid peroxidation status, serum hormone, metabolite, and mineral concentrations of Japanese quails reared under heat stress (34 °C). Int. J. Vitam. Nutr. Res..

[54] Deng R., Chow T.J. (2010). Hypolipidemic, antioxidant, and antiinflammatory activities of microalgae Spirulina. Cardiovasc. Ther..

[55] Alwaleed E.A., El-Sheekh M., Abdel-Daim M.M., Saber H. (2021). Effects of Spirulina platensis and Amphora coffeaeformis as dietary supplements on blood biochemical parameters, intestinal microbial population, and productive performance in broiler chickens. Environ. Sci. Pollut. Res..

[56] Park J., Lee S., Kim I. (2018). Effect of dietary Spirulina (Arthrospira) platensis on the growth performance, antioxidant enzyme activity, nutrient digestibility, cecal microflora, excreta noxious gas emission, and breast meat quality of broiler chickens. Poult. Sci..

[57] Khosravinia H. (2016). Mortality, production performance, water intake and organ weight of the heat stressed broiler chicken given savory (*Satureja khuzistanica*) essential oils through drinking water. J. Appl. Anim. Res..

[58] Nagaraju R., Reddy B., Gloridoss R., Suresh B., Ramesh C. (2014). Effect of dietary supplementation of humic acids on performance of broilers. Indian J. Anim. Sci..

[59] Pistova V., ARPÁŠOVÁ H., HRNČÁR C. (2016). The effect of the humic acid and garlic (*Allium sativum* L.) on performance parameters and carcass characteristic of broiler chicken. J. Cent. Eur. Agric..

[60] Ozturk E., Coskun I. Effects of humic acids on broiler performance and digestive tract traits. Proceedings of the Book of Abstracts of the 57th Annual Meeting of the European Association for Animal Production.

[61] Islam K., Schuhmacher A., Aupperle H., Gropp J. (2008). Fumaric acid in broiler nutrition: A dose titration study and safety aspects. Int. J. Poult. Sci.

[62] El-kelawy M., Elnaggar A.S., Enass A.E.-k. (2024). The influence of supplementing broiler chickens with humic acid or biochar as natural growth promoters on their productive performance, nutrient digestibility, and physiological performance. Egypt. Poult. Sci. J..

[63] Aristimunha P.C., Mallheiros R., Ferket P.R., Cardinal K.M., Moreira Filho A.L.d.B., Santos E., Cavalcante D.T., Ribeiro A.M.L. (2020). Effect of dietary organic acids and humic substance supplementation on performance, immune response and gut morphology of broiler chickens. J. Appl. Poult. Res..

[64] Mendiola J., Jaime L., Santoyo S., Reglero G., Cifuentes A., Ibañez E., Señoráns F. (2007). Screening of functional compounds in supercritical fluid extracts from *Spirulina platensis*. Food Chem..

[65] Tavernari F.D.C., Roza L., Surek D., Sordi C., Silva M., Albino L., Migliorini M., Paiano D., Boiago M. (2018). Apparent metabolisable energy and amino acid digestibility of microalgae Spirulina platensis as an ingredient in broiler chicken diets. Br. Poult. Sci..

[66] Agustini T.W., Suzery M., Sutrisnanto D., Ma’ruf W.F. (2015). Comparative study of bioactive substances extracted from fresh and dried Spirulina sp. Procedia Environ. Sci..

[67] Kharde S., Shirbhate R., Bahiram K., Nipane S. (2012). Effect of Spirulina supplementation on growth performance of broilers. Indian J. Vet. Res..

[68] Abdel-Moneim A.-M.E., Shehata A.M., Mohamed N.G., Elbaz A.M., Ibrahim N.S. (2022). Synergistic effect of Spirulina platensis and selenium nanoparticles on growth performance, serum metabolites, immune responses, and antioxidant capacity of heat-stressed broiler chickens. Biol. Trace Elem. Res..

[69] Chen H., Dong X., Yao Z., Xu B., Zhen S., Li C., Li X. (2012). Effects of prechilling parameters on water-holding capacity of chilled pork and optimization af prechilling parameters using response surface methodology. J. Anim. Sci..

[70] Ranjbarinasab Z., Mazhari M., Esmaeilipour O., Shahdadi F., Barazandeh A. (2024). Effects of the encapsulation of Lactobacillus acidophilus and Spirulina platensis on carcass yield and meat quality of broilers under heat stress conditions. Span. J. Agric. Res..

[71] Deng Y., Rosenvold K., Karlsson A., Horn P., Hedegaard J., Steffensen C., Andersen H.J. (2002). Relationship between thermal denaturation of porcine muscle proteins and water-holding capacity. J. Food Sci..

[72] Zhang Z., Jia G., Zuo J., Zhang Y., Lei J., Ren L., Feng D. (2012). Effects of constant and cyclic heat stress on muscle metabolism and meat quality of broiler breast fillet and thigh meat. Poult. Sci..

[73] El-Bahr S., Shousha S., Shehab A., Khattab W., Ahmed-Farid O., Sabike I., El-Garhy O., Albokhadaim I., Albosadah K. (2020). Effect of dietary microalgae on growth performance, profiles of amino and fatty acids, antioxidant status, and meat quality of broiler chickens. Animals.

[74] Onasanya G.O., Oke F.O., Sanni T.M., Muhammad A.I. (2015). Parameters influencing haematological, serum and bio-chemical references in livestock animals under different management systems. Open J. Vet. Med..

[75] Passantino L., Massaro M., Jirillo F., Di Modugno D., Ribaud M., Di Modugno G., Passantino G., Jirillo E. (2007). Antigenically activated avian erythrocytes release cytokine-like factors: A conserved phylogenetic function discovered in fish. Immunopharmacol. Immunotoxicol..

[76] Ipek H., Avci M., Iriadam M., Kaplan O., Denek N. (2008). Effects of humic acid on some hematological parameters, total antioxidant capacity and laying performance in Japanese quails. Eur. Poult. Sci..

[77] Chełmońskiego J. (2012). The effect of humic-fatty acid preparation on production parameters and meat quality of growing rabbits. Ann. Anim. Sci..

[78] Vaskova J., Patlevič P., Žatko D., Marcinčák S., Vaško L., Krempaská K., Nagy J. (2018). Effects of humic acids on poultry under stress conditions. Slov. Vet. Res..

[79] Khan Z., Bhadouria P., Bisen P. (2005). Nutritional and therapeutic potential of Spirulina. Curr. Pharm. Biotechnol..

[80] Kolluri G., Marappan G., Yadav A.S., Kumar A., Mariappan A.K., Tyagi J.S., Rokade J.J., Govinthasamy P. (2022). Effects of Spirulina (*Arthrospira platensis*) as a drinking water supplement during cyclical chronic heat stress on broiler chickens: Assessing algal composition, production, stress, health and immune-biochemical indices. J. Therm. Biol..

[81] Spínola M.P., Mendes A.R., Prates J.A. (2024). Chemical composition, bioactivities, and applications of Spirulina (*Limnospira platensis*) in food, feed, and medicine. Foods.

[82] Yahav S. (1999). The effect of constant and diurnal cyclic temperatures on performance and blood system of young turkeys. J. Therm. Biol..

[83] Flees J., Rajaei-Sharifabadi H., Greene E., Beer L., Hargis B.M., Ellestad L., Porter T., Donoghue A., Bottje W.G., Dridi S. (2017). Effect of Morinda citrifolia (noni)-enriched diet on hepatic heat shock protein and lipid metabolism-related genes in heat stressed broiler chickens. Front. Physiol..

[84] Duchateau A., de Thonel A., El Fatimy R., Dubreuil V., Mezger V. (2020). The “HSF connection”: Pleiotropic regulation and activities of Heat Shock Factors shape pathophysiological brain development. Neurosci. Lett..

[85] Liu S., Liu Y., Bao E., Tang S. (2024). The Protective Role of Heat Shock Proteins against Stresses in Animal Breeding. Int. J. Mol. Sci..

[86] Lei L., Yu J., Bao E. (2009). Expression of heat shock protein 90 (Hsp90) and transcription of its corresponding mRNA in broilers exposed to high temperature. Br. Poult. Sci..

[87] Bagatell R., Paine-Murrieta G.D., Taylor C.W., Pulcini E.J., Akinaga S., Benjamin I.J., Whitesell L. (2000). Induction of a heat shock factor 1-dependent stress response alters the cytotoxic activity of hsp90-binding agents. Clin. Cancer Res..

[88] Jackson S.E. (2012). Hsp90: Structure and Function. Molecular Chaperones.

[89] Lara L.J., Rostagno M.H. (2013). Impact of heat stress on poultry production. Animals.

[90] Kamel N.N., Ahmed A.M., Mehaisen G.M., Mashaly M.M., Abass A.O. (2017). Depression of leukocyte protein synthesis, immune function and growth performance induced by high environmental temperature in broiler chickens. Int. J. Biometeorol..

[91] Ohtsu H., Yamazaki M., Abe H., Murakami H., Toyomizu M. (2015). Heat stress modulates cytokine gene expression in the spleen of broiler chickens. J. Poult. Sci..

[92] Yu D., Rao S., Tsai L.M., Lee S.K., He Y., Sutcliffe E.L., Srivastava M., Linterman M., Zheng L., Simpson N. (2009). The transcriptional repressor Bcl-6 directs T follicular helper cell lineage commitment. Immunity.

[93] Herrero C., Hu X., Li W.P., Samuels S., Sharif M.N., Kotenko S., Ivashkiv L.B. (2003). Reprogramming of IL-10 activity and signaling by IFN-γ. J. Immunol..

[94] Dunkelberger J.R., Song W.-C. (2010). Complement and its role in innate and adaptive immune responses. Cell Res..

[95] Saurabh S., Sahoo P. (2008). Lysozyme: An important defence molecule of fish innate immune system. Aquac. Res..

[96] Ryan G. (2010). CD3 conformation is crucial for signalling. Nat. Rev. Immunol..

[97] Tedder T.F., Engel P. (1994). CD20: A regulator of cell-cycle progression of B lymphocytes. Immunol. Today.

[98] Hirakawa R., Nurjanah S., Furukawa K., Murai A., Kikusato M., Nochi T., Toyomizu M. (2020). Heat stress causes immune abnormalities via massive damage to effect proliferation and differentiation of lymphocytes in broiler chickens. Front. Vet. Sci..

[99] Song Z., Cheng K., Zheng X., Ahmad H., Zhang L., Wang T. (2018). Effects of dietary supplementation with enzymatically treated Artemisia annua on growth performance, intestinal morphology, digestive enzyme activities, immunity, and antioxidant capacity of heat-stressed broilers. Poult. Sci..

[100] Preynat-Seauve O., Coudurier S., Favier A., Marche P.-N., Villiers C. (2003). Oxidative stress impairs intracellular events involved in antigen processing and presentation to T cells. Cell Stress Chaperones.

[101] Kumar R., Sharma V., Das S., Patial V., Srivatsan V. (2023). *Arthrospira platensis* (Spirulina) fortified functional foods ameliorate iron and protein malnutrition by improving growth and modulating oxidative stress and gut microbiota in rats. Food Funct..

[102] Li J., Zhang Y., Yang S., Lu Z., Li G., Liu J., Zhou B., Wu D., Wang L. (2021). Isolation, purification, characterization, and immunomodulatory activity analysis of α-glucans from *Spirulina platensis*. ACS Omega.

[103] Beutler B. (2004). Innate immunity: An overview. Mol. Immunol..

[104] Zeweil H., Abaza I.M., Zahran S.M., Ahmed M.H., AboulEla H.M., Saad A.A. (2016). Effect of Spirulina platensis as dietary supplement on some biological traits for chickens under heat stress condition. Asian J. Biomed. Pharm. Sci.

[105] Disetlhe A., Marume U., Mlambo V., Dinev I. (2017). Humic acid and enzymes in canola-based broiler diets: Effects on bone development, intestinal histomorphology and immune development. S. Afr. J. Anim. Sci..

[106] El-Ratel I.T., El Basuini M.F., Khattab A.A., Mekawy A.I., Fouda S.F. (2023). Ameliorative impacts of sodium humate on heat-stressed laying Japanese quail (*Coturnix coturnix* Japonica). J. Anim. Physiol. Anim. Nutr..

[107] Cetin E., Guclu B.K., Cetin N. (2011). Effect of dietary humate and organic acid supplementation on social stress induced by high stocking density in laying hens. J. Anim. Vet. Adv..

[108] Gao J., Zhang H., Yu S., Wu S., Yoon I., Quigley J., Gao Y., Qi G. (2008). Effects of yeast culture in broiler diets on performance and immunomodulatory functions. Poult. Sci..

[109] Zeitz J., Fennhoff J., Kluge H., Stangl G., Eder K. (2015). Effects of dietary fats rich in lauric and myristic acid on performance, intestinal morphology, gut microbes, and meat quality in broilers. Poult. Sci..

[110] Yamauchi K.-e. (2002). Review on chicken intestinal villus histological alterations related with intestinal function. J. Poult. Sci..

[111] López-García Y.R., Gómez-Rosales S., Angeles M.d.L., Jiménez-Severiano H., Merino-Guzman R., Téllez-Isaias G. (2023). Effect of the addition of humic substances on morphometric analysis and number of goblet cells in the intestinal mucosa of broiler chickens. Animals.

[112] Mamashli M., Ghorbani B. (2025). Intestinal mucosal morphology and microbial flora population in Arianstrain broilers fed with Spirulina platensis supplemented drinking water. J. Poult. Sci. Avian Dis..

[113] Rostagno M.H. (2020). Effects of heat stress on the gut health of poultry. J. Anim. Sci..

